# Rapid diagnostic methods for SARS-CoV-2 (COVID-19) detection: an evidence-based report

**DOI:** 10.25122/jml-2021-0168

**Published:** 2021

**Authors:** Manish Kumar Verma, Parshant Kumar Sharma, Henu Kumar Verma, Anand Narayan Singh, Desh Deepak Singh, Poonam Verma, Areena Hoda Siddiqui

**Affiliations:** 1. Department of Biochemistry, G.S.V.M. Medical College, Kanpur, India; 2. Department of Electronic Engineering, Kwangwoon University, Nowon-gu, Seoul, South Korea; 3. Department of Immunopathology, Institute of lungs Biology and Disease, Comprehensive Pneumology Center, Munich, Germany; 4. Amity Institute of Biotechnology, Amity University Rajasthan, Jaipur, India; 5. Department of Biotechnology, IFTM University, Moradabad, India; 6. Department of Laboratory Medicine, Sahara Hospital, Viraj Khand, Gomti Nagar, Lucknow, India

**Keywords:** COVID-19, SARS-CoV-2, sensor chip-based, Point-of-Care Testing, diagnostics

## Abstract

Since December 2019, the severe acute respiratory syndrome coronavirus-2 (SARS-CoV-2) has been a global health concern. The transmission method is human-to-human. Since this second wave of SARS-CoV-2 is more aggressive than the first wave, rapid testing is warranted to use practical diagnostics to break the transfer chain. Currently, various techniques are used to diagnose SARS-CoV-2 infection, each with its own set of advantages and disadvantages. A full review of online databases such as PubMed, EMBASE, Web of Science, and Google Scholar was analyzed to identify relevant articles focusing on SARS-CoV-2 and diagnosis and therapeutics. The most recent article search was on May 10, 2021. We summarize promising methods for detecting the novel Coronavirus using sensor-based diagnostic technologies that are sensitive, cost-effective, and simple to use at the point of care. This includes loop-mediated isothermal amplification and several laboratory protocols for confirming suspected 2019-nCoV cases, as well as studies with non-commercial laboratory protocols based on real-time reverse transcription-polymerase chain reaction and a field-effect transistor-based bio-sensing device. We discuss a potential discovery that could lead to the mass and targeted SARS-CoV-2 detection needed to manage the COVID-19 pandemic through infection succession and timely therapy.

What is new/important. Learning Points

• The use of the electrochemical and serological sensors for SARS-CoV-2 diagnosis;
• Sensitivity enhancement compared to the traditional diagnostic devices;
• State-of-the-art SARS-CoV-2 diagnosis strategies such as miniaturized electrochemical analyzers;
• AI-supporting smartphone-based operations for rapid diagnosis;
• Evidence on the diagnostic accuracy of all known tests for SARS-CoV-2, as well as tests for antibodies to SARS-CoV-2;
• Most qPCR assays have three targets: Orf1, E and N genes.
• Manufacturers can apply for emergency use authorization (EUA) from the United States Food and Drug Administration for clinical diagnostic use.
• Rapid antigen detection has the potential to become an important tool for the early diagnosis of SARS-CoV-2, particularly in situations with limited access to molecular methods.

## Introduction

**Figure 1.**
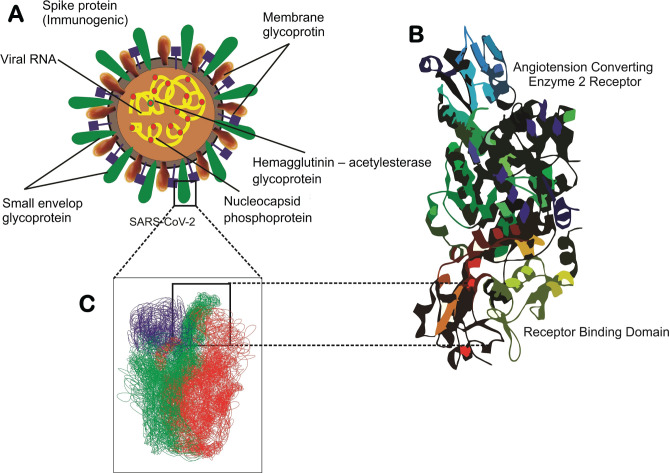
The structure of SARS-CoV-2 virus binding to the ACE2 host receptor [3]. (A) Schematic SARS-CoV-2 binding to the ACE2 host receptor. Schematic of a SARS-CoV-2 particle, an enveloped ssRNA virus expressing the spike glycoprotein (S) that mediates the binding host cells at its surface. (B) Structural studies have previously obtained a complex between the receptor-binding domain (RBD, a subunit of the S glycoprotein) and the angiotensin-converting enzyme 2 (ACE2) receptor. (C) Schematic of probing SARS-CoV-2 binding using atomic force microscopy (AFM). The initial attachment of SARS-CoV-2 to cells involves specific binding between the viral S glycoprotein and the cellular receptor, ACE2.

In December 2019, the novel coronavirus disease 2019 (COVID-19) was first reported and diagnosed in Wuhan, China. Coronavirus (CoV) is a member of the Coronavirinae subclass of the Coronaviridae family of the order Nidovirales. These viruses are members of the coronavirus subgenera alpha, beta, gamma, and delta [1]. These viruses are typically 150–160 nm in size and classified as envelope-type viruses [2]. The virus structure comprises unfermented positive ss-RNA, a nucleoprotein capsid suspended in a matrix, and a Spike protein (S). The virus' fully labeled structure is shown in Figure 1.

After comparing DNA viruses with RNA viruses, it was discovered that the substitutions per nucleotide site per cell infection ranges between 10–8 to 10–6 (s/n/c) and 10–6 and 10–4 (s/n/c), respectively [4]. Genome sequencing was done regularly to identify genetic changes in SARS-COV-2, but the infection is difficult to diagnose and treat [5, 6].

After five days, the first symptom of COVID-19 appears. The onset of the first symptom can range from sixth to the 41^st^ day, with an average of 14 days. The onset of symptoms is determined by the infected patient's age and immune system. According to studies, it takes less time for patients over 70 years to recover than for younger patients [7, 8]. Fever, dry cough, dyspnea, myalgia or fatigue, headache, hemoptysis, and diarrhea are some of the most common symptoms of COVID-19 [9]. Patients infected with this virus can suffer potential damage to other vital organs, such as the gastrointestinal, cardiac, renal, and nervous systems [10].

Although some patients do not have a fever, they suffer from acute respiratory distress syndrome (ARDS) and acute organ injury. One of the significant symptoms of COVID-19 is that patients lose their ability to smell and taste [11]. It was proven that convalescent plasma transfusion (CPT) is useful in patients with severe COVID-19 [12].

The presence of protein found in the virus was discovered to be the primary cause of this disease, contributing to the development of diagnostic methods [13]. SARS-CoV2 comes in two variants, both of which are quite stable.

Further, it can be detected in aerosols for up to 3 hours, on cardboard for up to 24 hours, and copper for up to 4 hours due to its high stability. Furthermore, it was discovered that SARS-CoV-1 and SARS-CoV-2 have longer viability on polypropylene and stainless steel materials than on cardboard or copper. They can survive on plastic and stainless-steel surfaces for up to 2–3 days. Researchers also demonstrated a similar half-presence in aerosols, which lasted an average of 2.7 hours and 13 hours on steel surfaces [14, 15].

According to a rapid report published by McIntyre in February 2020, the virus has spread to a level of pandemic potential comparable to influenza, with an R0 of 2.2 [16]. COVID-19 was declared a global pandemic on March 11, 2021. At the moment of writing, 145,501,934 confirmed cases were reported across 221 countries, with a death rate of more than 2,992,193 lakhs people reported to the World Health Organization (WHO). Currently, it is reported and observed in 221 countries and regions across the world. (https://www.worldometers.info/coronavirus/coronavirus-death-toll/).

As stated earlier, due to the evident presence of a particular protein in the virus, it was an indicator in many diagnostic tools for identification in respiratory samples. Several labs conducted tests to detect this protein's presence in respiratory specimens. The Centers for Disease Control and Prevention (CDCP) and WHO, as well as the Indian Council of Medical Research (ICMR), issued specific guidelines for the transportation and handling of infected patients’ samples. In this section, we will go over sample transportation and testing methods developed by various companies for early detection, which will aid in disease containment.

### Pre-analytical issues

#### Specimen collection and transport

Samples from the nasopharyngeal or pharyngeal region are collected and transported to testing laboratories at a minimum temperature of 4°C. They can be stored at -70°C in the lab for more than five days. As recommended by CDC, the current diagnostic approach includes collecting samples from the patient's sputum, blood/serum, and a swab from the nasopharyngeal and or pharyngeal swab, bronchoalveolar lavage, tracheal aspirate, nasopharyngeal aspirate, or nasal wash. For swab-based SARS–CoV-2 testing, a nasopharyngeal specimen is commonly preferred over other swabs.

However, other swab samples such as oropharyngeal, mid-turbinate, or anterior nares are also acceptable and can be considered for identification [17, 18]. Sputum, endotracheal aspirate, and bronchoalveolar lavage samples were more sensitive [19].

#### Emergence and genome Evolution of SARS-CoV-2 Virus

More viral genomes have been sequenced and added to the previously known category as the SARS-CoV-2 or COVID-19 pandemic has progressed. While phylogenetic and phylogeographic inferences can be used to predict and remise the disease, some general health experts in Wuhan made some historical findings by identifying the disease as the leading group of pneumonia cases [20]. The highly compensated infection rate of virus replication within the hosts compensates for the recommended lower mutation rates.

Although no evidence of the virus's ability to mutate has previously been discovered, it will cause extreme changes in any phenotype, such as transmissibility and virulence [21], so it is critical to screen for any changes in phenotypic changes as the infection spreads. Any decrease in the number of positive cases and the Case Fatality Ratio (CFR) of COVID-19 will undoubtedly be due to rising resistance in the human population and an epidemiological environment hostile to mutational changes in the viral structure.

#### Genomic features of SARS-CoV-2

**Figure 2.**
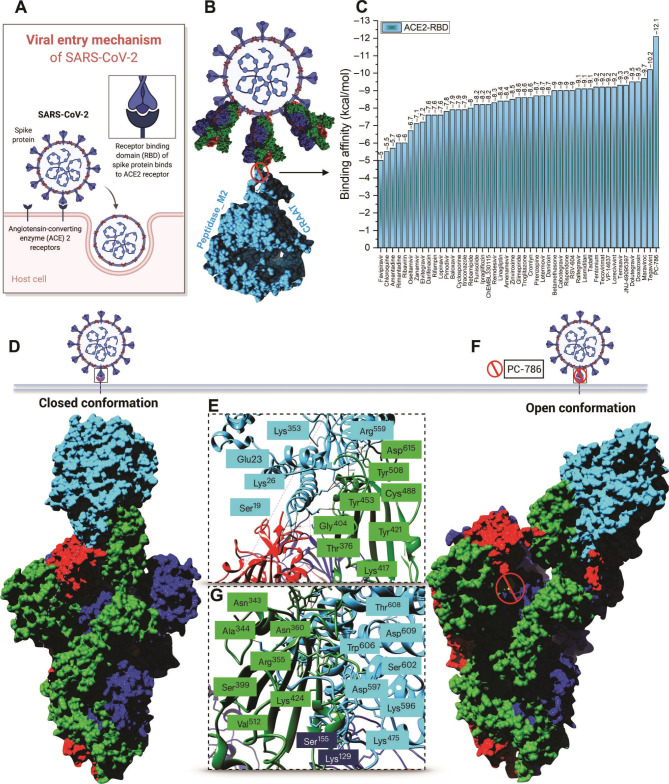
Schematically representation of the RBD-ACE2 complex protein-protein interaction. (A) Viral entry mechanism of SARS-CoV-2. (B) Trimeric S protein RBD interaction inhibition with ACE2 by repurposed antiviral drugs. (C) Bar graph of binding affinities (kcal/mole) of selected antiviral drugs from virtual screening to RBD-ACE2 complex. (D) ACE2 binding to trimeric S protein RBD in a closed conformation. (E and G) Key residues of the interaction mechanism. Blue-colored residues are from the ACE2 enzyme and green-shaded residues from trimeric S protein (F) Open conformation of the antiviral drug (PC786) that conjugates the RBD-ACE2 complex [4]. (Reproduced with permission, © AAAS Science Advances).

Based on structural studies [22, 23] and biochemical experiments [24, 25], there are two types of notable genome features of SARS-CoV-2: alpha and beta types. SARS-CoV-2 can be optimized by binding receptor Angiotensin-Converting Enzyme 2 (ACE2); and SARS-CoV-2 spike protein has a useful polybasic cleavage site at the S1–S2, which limits the inclusion of 12 nucleotides and also prompts the anticipated security of three O-linked glycans around as shown in (Figure 2).

#### SARS-CoV-2 origin and related theories

It is quite hypothetical that SARS-CoV-2 emerged from laboratory manipulation of SARS-CoV-like coronavirus in 2019. As noted over, the receptor-binding domain (RBD) of SARS-CoV-2 is upgraded for authoritative to ACE2 of human cells [26]. Betacoronavirus consists of A, B, C and D subgroups; SARS-CoV and SARS-CoV-2 come under the B subgroup [27].

In any case, hereditary information verifiably shows that SARS-CoV-2 is not received from any recently used infection backbone [28]. Based on the analysis of subgroup B, key insertions and deletions were identified and named M1 to M6 in the ORF3a, M, ORF7a, 7b, 8 and N genes, respectively [28].

## Current Diagnostic Tests for COVID-19

### Target selection for the rapid detection of COVID-19

**Figure 3.**
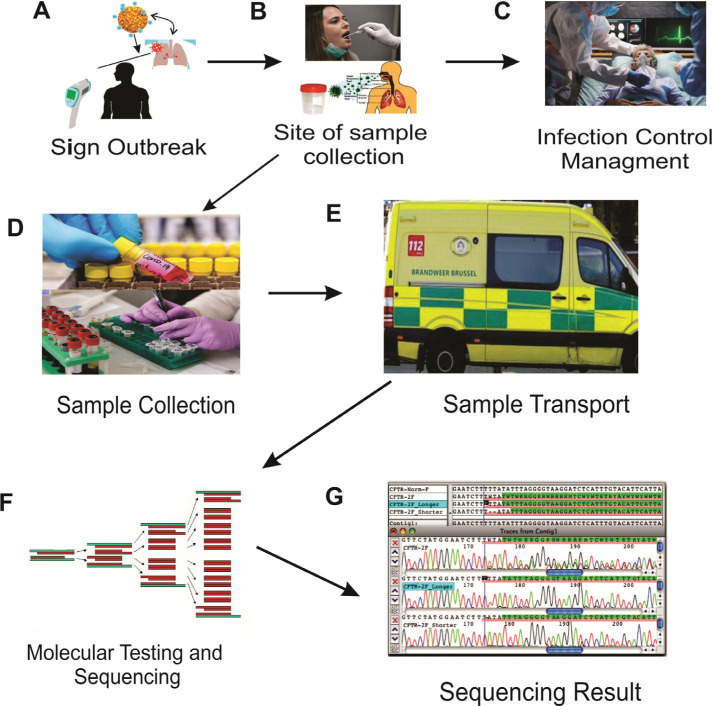
Sample collection process from people and its handling. Process of post-analytical flow. (A) Schematic representation of a human affected by the SARS-Cov-2 infection. (B) Nasopharyngeal and or pharyngeal swab sample collection. (C) Severe cases are admitted to the hospital, and mild cases are self-quarantined at home. (D) Sample collection tube in a viral transport medium. (E) Sample transport and maintenance at 4°C. (F) Measure sequence and cycle threshold. (G) Interpretation of the positive or negative result.

The detection of SARS-CoV-2 RNA was demonstrated for the use of early coronavirus disease detection. It could be useful in controlling wellspring contamination and preventing patients from being infected with the virus. Detecting coronaviruses quickly and precisely is now becoming extremely important. With advancements in atomic science, innovation, and nucleic corrosive location techniques, it was possible to find a solution for detection of techniques for a rapidly growing pandemic through progressive viral detection innovation. Polymerase chain reaction (PCR)-based detection is characterized as a fast recognition, high sensitivity, and specificity detection technique, and it has been regarded as the “gold standard” for virus detection. Several other molecular tests compete to recognize coronavirus RNA, but they are not based on PCR. Here, we evaluate and the various methodologies available for coronavirus detection to help future scientists combat such a pandemic by developing a novel, fast, and precise detection method. The current diagnostic workflow for COVID-19 is described in (Figure 3).

The most promoted technique is the real-time reverse transcription-polymerase chain reaction (RT-PCR) test strategy [29, 30]. The initial positive nucleic acid test and the lung changes seen on computed tomography (CT) scans must be compared and checked. Positive cases typically have a low lung CT, which is similar to a reverse halo sign, but this should be supported by several other nucleic acid tests [31, 32].

The detection of nucleic acids, on the other hand, has its limitations in terms of ease of operation, time efficiency, and degree of pollution. However, CT results can differ between infected patients and have low specificity. It is also recommended to test for the presence of IgM/IgG antibodies. Because of its high sensitivity, the enzyme-linked immunosorbent assay (ELISA) test is used [33]. SARS-CoV N-based IgG ELISA has a 94.7% sensitivity, SARS-CoV S-based IgG ELISA has a 58.9% sensitivity, and SARS-CoV-2 IgG/IgM are still being studied [34]. Table 1 shows the current COVID-19 detection methods that are available.

**Table 1.** Current diagnosis method available for COVID-19.

**Method available**	**Working principle**	**Sample Source**	**Advantage**	**Time required**	**Disadvantage**
**Next generation sequencing (NGS)**	Gross genome sequencing	Oropharyngeal swabs or nasopharyngeal swabs, sputum, lower respiratory tract aspirates, broncho-alveolar lavage	Eminently sensitive and distinct, able to identify newfangled strain	1–2 day	Recommended with highly equipped lab and needs high expertise of the lab technician and sophisticated lab equipment
**RT-PCR**	Unique probed-primer based detection	Oropharyngeal swabs or nasopharyngeal swabs, sputum, lower respiratory tract aspirates, broncho-alveolar lavage	Higher Response time needs slight amount of DNA sample, can be applicable in a single step, traditional technique in viral diagnostics	3–4 hrs.	Needs expensive lab equipment, complex process with time consuming steps
**Reverse transcription loop-mediated isothermal amplification (RT-LAMP)**	Detection based on more than two sets of specific primers	Oropharyngeal swabs or nasopharyngeal swabs, sputum, lower respiratory tract aspirates, broncho-alveolar lavage	Highly accurate and repeatable in fixed climate conditions	1 hr.	Highly sensitive and too prone to cross contamination
**Serological (Traditional antibody test)**	Antigen/Antibodies IgM/IgG	Oropharyngeal swabs or nasopharyngeal swabs, sputum, lower respiratory tract aspirates, broncho-alveolar lavage	Highly Selective and Sensitive	4–6 hrs.	Highly time taking process with 3-4 days incubation and testing time
**Rapid serological**	Antigen/Antibodies IgM/IgG	Oropharyngeal swabs or nasopharyngeal swabs, sputum, lower respiratory tract aspirates, broncho-alveolar lavage	Applicable for Point-of-care testing (POCT)	15–30 mins	Highly time-consuming process with 3-4 days for incubation and testing time; too prone to cross contamination
**CT Scan**	Chest images	Based on human physiology	Highly sensitive and selective if the results are combined with RT-PCR findings	1 hr.	Predictability from other viral pneumonias and abnormal CT hysteresis
**Virus Isolation**	In vitro live virus isolation and propagation	Oropharyngeal swabs or nasopharyngeal swabs, sputum, lower respiratory tract aspirates, broncho-alveolar lavage	Highly (100%) specific. Gold standard	5–15 days	Less sensitive if isolation is not prompt

## Diagnosis of SARS-COV-2 with Molecular and Chip-Based

### Reverse transcription-polymerase chain reaction (RT-PCR)

RT-PCR is now widely used to detect COVID-19 in respiratory secretions [35]. However, it has some drawbacks, such as biological contamination risks due to inappropriate handling of patient samples, the need for well-equipped laboratories and skilled personnel, and the long time required for the expected results [36]. RT-PCR-based kits have recently been developed for the qualitative detection of viral infection in various biological fluids such as alveolar lavage fluid, nasopharyngeal swabs, sputum, and blood [37]. The Chinese Center for Disease Control and Prevention recommended that ORF1ab and N-gene regions must be used for COVID-19 detection by RT-qPCR. The use of RT-qPCR-based techniques (Taq Man-based fluorescence signal) to determine ORF1ab and N-gene regions in respiratory samples was well described, and results were reported [37].

Wang *et al.* conducted a study using RT-qPCR, which revealed a 91.7% positive rate in saliva samples from SARS-CoV-2 infected patients. Saliva has been proposed as a promising non-invasive patient sample for disease diagnosis and monitoring [38]. COVID 19 tests, in general, have lower accuracy and sensitivity than RT-PCR-based tests because they do not amplify small amounts of target viral RNA. Furthermore, the assurance of novel coronavirus SARS-CoV-2 has specifically designed primers to be used [39]. However, while RT-PCR-based methodologies have some advantages over other tests, they also have some disadvantages, such as the fact that results can take anywhere from a few hours to up to two days [40].

Furthermore, RT-PCR necessitates well-trained personnel to handle and go through complicated sample processing methods. These limitations have an impact on its functionality and widespread application in the management of antiviral agents. Several Clinical Laboratory Improvement Amendments (CLIA) have recently waived the molecular tests that have been reported for point-of-care utilization (https://www.cdc.gov/flu/professionals/diagnosis/molecular-assays.html).

### Reverse transcription loop-mediated isothermal amplification (RT-LAMP)

**Figure 4.**
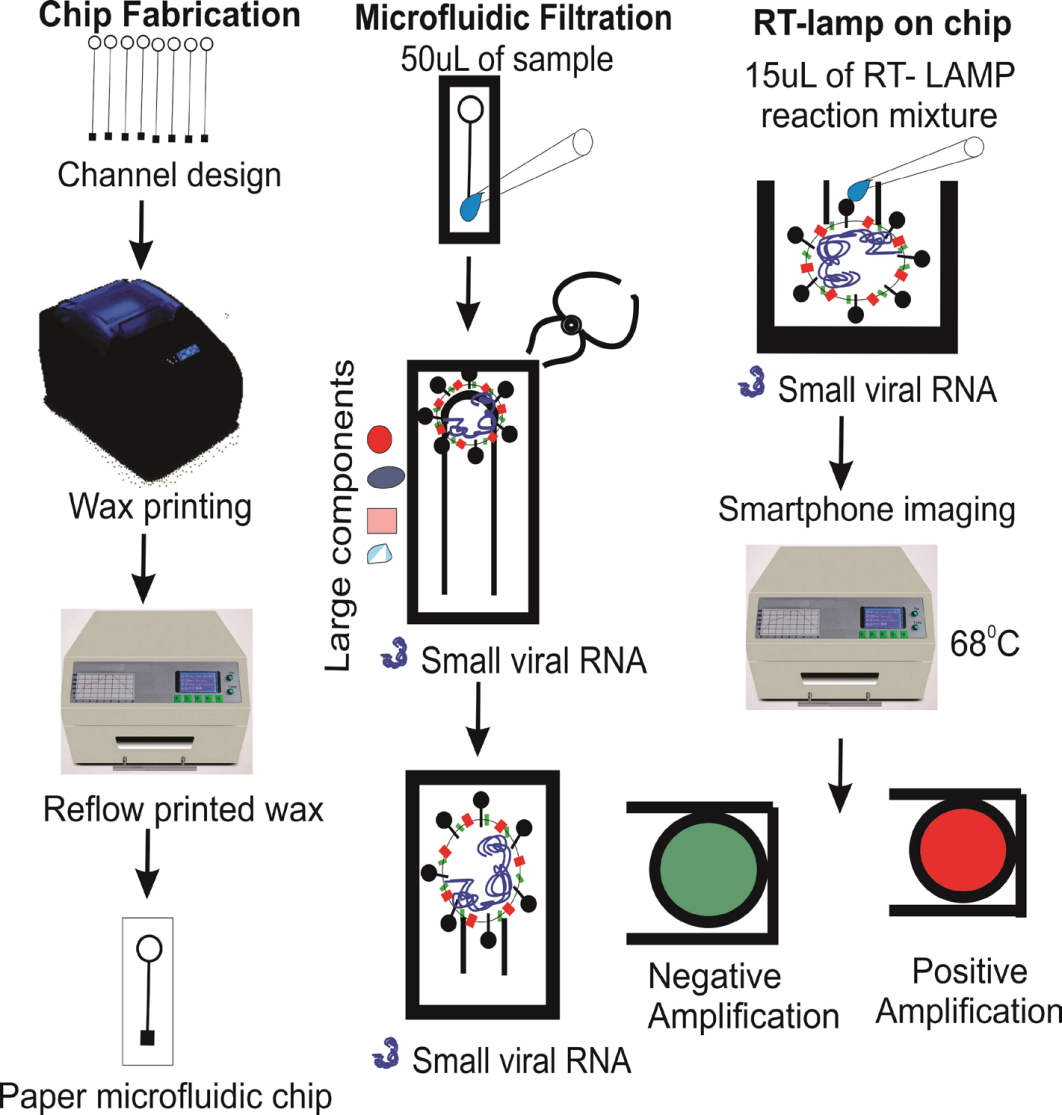
Schematically representation of development stages of paper microfluidic RT-LAMP assay: (A) Development of paper microfluidic chips. (B) Loading of ZIKV-spiked samples, (C) Amplification step and color change observation via smartphone [41].

Notomi *et al.* developed the reverse transcription loop-mediated isothermal amplification (RT-LAMP) assay for the first time in 2000 [42]. It is a sensitive, fast method with simple operation, command over the visual nucleic acid amplification method, and simple results interpretation. This technique has recently been used to detect pathogenic infections like SARS-CoV-2, influenza, Middle East Respiratory Syndrome (MERS)-CoV, West Nile, Ebola, Zika, yellow fever, and others (Figure 4) [43–48].

**Figure 5.**
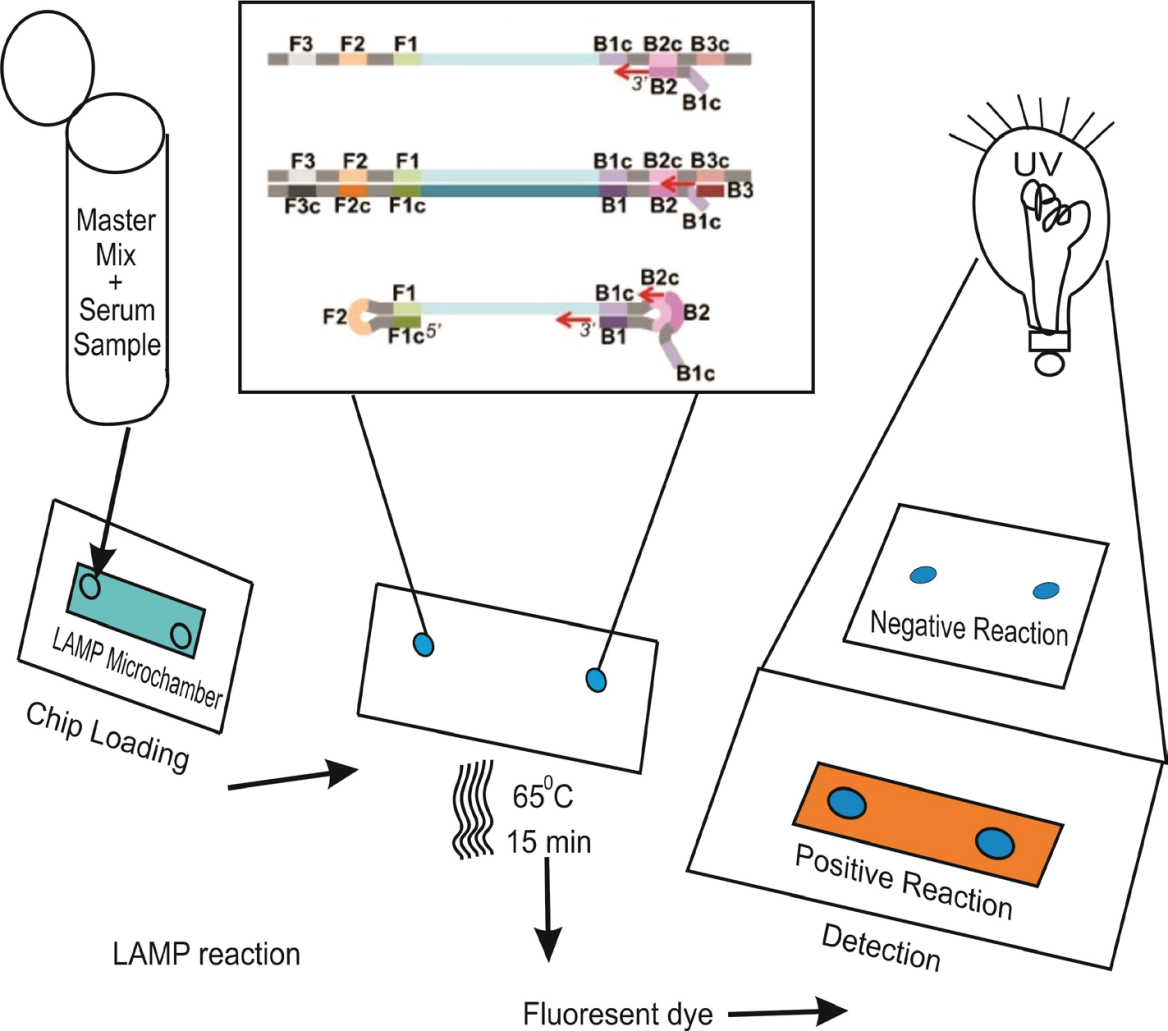
LAMP reaction steps on micro chamber via interaction of a fluorescent dye [51].

According to the reports, six primers were used on an isothermal LAMP method called the isothermal LAMP-based method for COVID-19 testing

(iLACO) to amplify a fragment of the ORF1ab gene [34]. The Basic Local Alignment Search Tool (BLAST) was used to compare 11 related viruses in this method. Although the developed system’s reaction time ranged from 15 to 40 minutes, its assays were initially valuable for testing COVID-19 positive patients with outbreak control due to their accuracy, simplicity, and versatility [49]. In this study, SARS-CoV-2 RNA was used in conjunction with the RT-LAMP method and reported for visual and colorimetric detection. Temperature is the most important factor for a specific and efficient RT-LAMP reaction. A conventional thermocycler was used to investigate three different temperatures, 65°C, 68°C, and 70°C (for maintaining one temperature required for RT-LAMP) (Figure 4). The authors tested the system on RNA samples purified from COVID-19 patients’ respiratory samples in Wuhan, China. Later in the study, the researchers concluded that this method could detect viruses without complex diagnostic materials [50]. RT-LAMP amplification, a polyester-toner (PeT) for visual detection on-chip, fluorescent DNA intercalator SYBR Green I was added for visual detection on-chip, as shown in Figure 5.

### Field-effect transistor-based biosensor for SARS-CoV-2 detection

**Figure 6.**
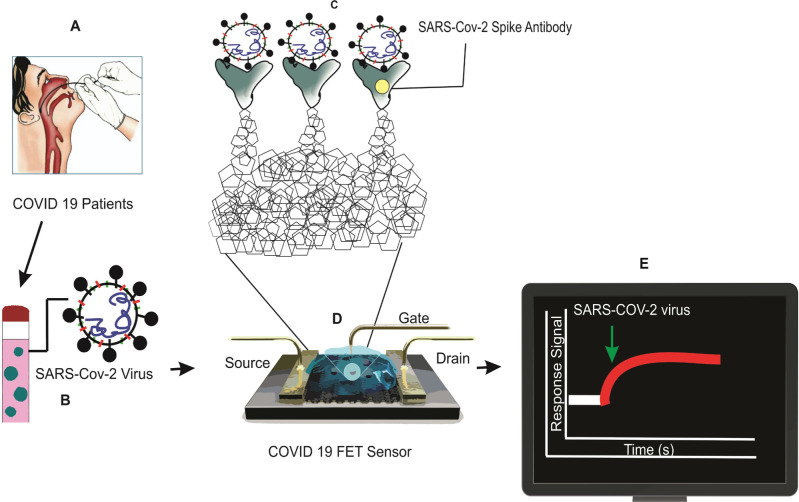
FET biosensor operation procedure for SARS-CoV-2 diagnosis [52].

Seo *et al.* discovered that the graphene-based field-effect transistor biosensor could detect SARS-CoV-2 from a human nasopharyngeal swab. The sensing layer of the biosensor was chosen to be graphene, and the SARS-CoV-2 spike antibody was conjugated onto the graphene sheet via a 1-pyrene butyric acid N-hydroxy succinimide ester linkage. At low levels of phosphate-buffered saline and clinical transport medium, this field-effect transistor (FET)-based biosensing device could determine the SARS-CoV-2 spike protein concentration. As a result, this biosensor has been successfully constructed as a promising FET biosensor for SARS-CoV-2 without the need for pretreatment or labeling [62]. The detailed procedure is depicted in Figure 6.

Graphene is implemented as a sensing material, and the SARS-CoV-2 spike antibody is conjugated onto the graphene sheet via 1-pyrene butyric acid N-hydroxysuccinimide ester, which is an interfacing molecule as a probe linker. Schematic diagram of COVID-19 FET sensor operation procedure: (A) Nasopharyngeal swab sample collection. (B) Sample collection tube in the viral transport medium. (C) Graphene-based biosensing device functionalized with the SARS-CoV-2 spike antibody. (D) The fabricated device through 1-pyrene butyric acid N-hydroxy succinimide ester (PBASE). (E) Response SARS-COV-2 signal on display.

## Radiological Features of COVID-19

### Computed tomography (CT)

CT is a non-invasive imaging technique that can detect certain trademark indications in the lung associated with the novel coronavirus SARS-CoV-2 [32, 53]. As a result, computed tomography can be an effective method for the early detection and screening for COVID-19 positive patients. Despite such advantages, computed tomography may share particular comparable imaging highlights between COVID-19 and various types of pneumonia, making it difficult to distinguish between them both. Radiological tests, particularly chest CT for COVID-19, have been developed for initial diagnosis and monitoring disease progression. A proficient and precise evaluation technique is required for the rapid detection technique using computed tomography assessments for meeting the requirements of accurate level detection of COVID-19 differentiating between pneumonia [54, 55]. In one study, a small number of patients were tested using RT-PCR analysis, which adhered to the gold standard. The researchers proposed that non-contrast chest computed tomography has a sensitivity of 98% for detecting COVID-19 disease, compared to the initial RT-PCR sensitivity, which was 71%. This study reported a lack of sensitivity at the start of RT-PCR testing, which was confirmed by another study [56]. It was revealed that 3% of patients had negative RT-PCR results for the COVID-19 virus regardless of chest CT outcomes. Due to various viral pneumonia, using a chest CT reduces the number of false-negative COVID-19 results. The results of an analysis of 121 patients report from four studies conducted in the early, transitional. Late stages of disease revealed that RT-PCR was more sensitive than CT scans and was proposed as a necessary supportive diagnostic method [57, 58]. In addition, the American Society for Radiation Oncology proposed developing CT-based diagnostic techniques for COVID-19 to improve SARS-CoV-2 detection rates [59].

## Point of Care Testing (POCT)

POCT allows for faster test results, potentially reducing the turnaround time of the results. At the moment, the majority of SARS-CoV2 confirmatory tests are based on a molecular diagnostic method that takes at least 24 hours to produce results. Because of a lack of POCT and other limited-resource settings, it has become a bottleneck, adding to the complications of the current pandemic outbreak situation. As a result, developing a POCT for SARS-CoV-2 has become critical. The ideal POCT should be more sensitive and specific for detecting the SARS-CoV2 virus in real-time while also being easy to use.

### Sensor-Based (Molecular, Serological and antigen tests) Testing approved in-vitro Diagnostics (IVDs)

#### SARS-CoV-2 Spike Protein ELISA Kit

The serological analysis is currently being developed [60, 61]. Serology testing (IgM and IgG) in human serum of SARS-CoV-2 patients is only used for observational purposes and not as a diagnostic tool. Rapid antigen horizontal stream examinations have the potential benefit of detecting the SARS-CoV-2 virus in a short period with minimal effort. Nevertheless, they are likely to experience the effects of low sensitivity as a result of the use of this technique for previous infections [62–64]. A significant challenge associated with the inconsistencies of COVID-19 patients is due to the viral load, and the antigen discovery may cause some missing cases attributed to low irresistible weight or testing changeability.

The serological analysis of the host reaction to contamination is a circuitous proportion of disease that must be handled cautiously. Serological strategies have been developed and have proven helpful in the epidemiology of COVID-19 [59, 65]. If the response is to establish IgG reactions, serology detection is unlikely to play a role in a dynamic case of the board. Cell cultures are not recommended for disease diagnosis or infection monitoring strategies to validate recent SARS-CoV-2 cases with their resistance rates [66].

#### Lateral-flow immunoassay (LFIA)

Rapid antibody recognition-based assays include the following tests: 1) Bio-medics rapid test and Sure screen rapid test cassette, 2) Gold site diagnostics kit, 3) Assay Genie rapid POC kit, and 4) Viva-Diag SARS-CoV-2 antibodies. DPP SARS-CoV-2 IgM/IgG is a POC test approved by The United States Food and Drug Administration (FDA) with a rapid LFIA test developed by Chembio Diagnostics that can produce results in 15 minutes using a finger pricked blood sample. This test is based on optical readout via micro reader 1 and 2 analyzers. Diazyme DZ-Lite SARS CoV-2 IgM and IgG chemiluminescence immunoassay (CLIA) Kits are another FDA and Emergency Use Authorization (EUA)-approved antibodies-based kit [67]. The DZ-Lite SARS CoV-2 IgM and IgG CLIA Kits are used in their method, based on a CLIA analyzer with a throughput of 50 tests/hour. Various CE-certified ELISA kits developed by in vitro diagnostics (IVD) manufacturers such as Euroimmun, IBL International, DRG Diagnostics GmbH, and Epitope Diagnostics are widely available on the market.

The majority of them are based on IgG and IgM antibodies in the patient’s blood. These assays are designed to measure and detect the presence of IgG and IgM in patient blood, serum, or plasma samples. It also includes a simple operation process in which the patient’s finger is pricked, and the blood is analyzed, with the resulting output similar to a pregnancy test, which takes approximately 10–15 minutes to complete. All of these are compatible with single-use disposable cartridges that can be stored at room temperature. The handling procedure is quite simple; it makes use of a simple, easy-to-follow procedure. Pipetting a few drops of blood from a finger prick or vein, for example, can be used. The blood sample flows through the strip, and the result of the sample can be interpreted in 5 to 10 minutes. These are single-use expendable cartridges that can be used at room temperature regularly.

The Indian Council of Medical Research (ICMR) and the All India Institute of Medical Sciences (AIIMS) Delhi have recently tested the COVID antigen from SD biosensors exclusively in India. According to the guidelines, these antigen tests are highly selective and specific, with sensitivity ranging from 55% to 84%. In India, it is almost mandatory that COVID-19 patients undergo this antigen testing. If found positive, RT-PCR testing is not required; however, if found negative, RT-PCR testing is required, and it takes 30 minutes to complete.

## New Diagnostic Modalities for SARS-COV-2 Detection

*Vivalytic COVID-19 Test*: Bosch, Germany, and Randox Laboratories, UK, created the Vivalytic SARS-COV-2 test. This kit’s test time is less than 2.5 hours. SARS-COV-2 NS and TS samples were used to detect multiple respiratory viruses with influenza A and B using a fully automated and rapid point-of-care molecular test [68]. *Abbott ID Now*™: Based on the RdRp gene of SARS-COV-2, this test kit can provide qualitative results in 5 minutes using NS and TS samples [68]. *Qwik Zyme*: This test is more sensitive and specific, as well as simple to use; it can even be performed outside of the clinical setup; the solution allows it to be used extensively for outside-of-lab testing, theoretically in any geographic region, which can help to reduce virus spread [85].

## Other Diagnostic Methods

*Xpert Xpress SARS CoV2*: The N2 gene is detected using a rapid point-of-care RT-PCR test. The test has a detection limit of 250 copies/mL and takes 45 minutes to complete. This type of testing is done with cartridges. The results are comparable to those obtained using RT-PCR. The test does not necessitate any specialized training or skilled personnel.

*Bio Fire*: This POCT from Biomereiux has a single place and multiplexed SARS-CoV-2 detection cartridges. This system is approved by Emergency Use Authorization, and it can detect respiratory pathogens in about 45 minutes.

*TRUENAT*: For COVID-19, it is a chip-based technology using RT-PCR technology developed by Molbio, a company based in India. In one hour, the results can be confirmed as positive. The kits have been approved by the Indian Council of Medical Research (ICMR). It is widely used at the district/community health center level in India. It has made a significant contribution to covering the widespread testing in India’s healthcare system. It includes an RNA extractor as well as a processor; also, it detects the ORF3a gene of COVID19 and the whole process takes 1 hour [69].

## State-of-Art COVID-19 Diagnostics Strategies

The most common technique identifying SARS-CoV2 diagnosis is real-time PCR. The RT-PCR test is also highly recommended for disease progression monitoring. Although RT-PCR is a sensitive and specific technique, it requires skilled personnel to perform the test correctly. A chest CT or X-ray must be performed for the COVID-19 patient suspected of having viral pneumonia. In addition, ELISA-based tests have recently been investigated for the specific detection of COVID-19 antibodies. Individuals with an active immune response to SARS-CoV-2 can be identified using serological assays. Micro-neutralization is a test used to detect specific antibodies in a serum sample; it is thought to be more sensitive and specific for neutralizing antibody analysis. The presence of antibodies indicates that the infected person has developed an immune response. Serology tests, on the other hand, are only recommended for observational purposes and not for diagnostic purposes. Point-of-care diagnostics (POCD) allows for faster test results, potentially enhancing active medical care. Currently, all available COVID-19 tests are hospital/laboratory-based, with a turnaround time of 24 to 48 hours. POCD inaccessibility and a scarcity of skilled personnel are also significant issues in the current pandemic. As a result, there is a pressing need to develop a COVID-19 POCD test that is simple to use, sensitive, specific, and cost-effective.

According to Kaushik *et al.*, electrochemical SARS-CoV-2 immunosensing is sensitive, and with the help of nano-enabled biosensors, we can detect SARS-CoV-2 at the picomolar level, even at point-of-care. It is recommended to adopt and optimize an immunosensing approach by Kaushik *et al.*, who developed an electrochemical SARS-CoV-2 biosensor. This method is also ideal for quick data analysis, secure data storage, and remote data sharing with healthcare professionals.

## Conclusions

The development of a rapid, robust, accurate, precise, and cost-efficient point-of-care sensor/device is critical in the event of a pandemic caused by SARS-CoV-2. It should be so simple to use in nursing homes, government healthcare facilities, and community settings that even a layperson could operate it. It should apply to a wide range of people, from the average citizen to highly skilled workers. The government will be able to control cases better and combat the pandemic if these rapid POC tests are implemented. Due to the shortage of research in this area, finding simple assays with high sensitivity and ease of use has become difficult. For SARS-CoV-2 detection, precise RT-LAMP assays are now preferred. In studies comparing RTPCR and the RT-LAMP assay for SARS-CoV-2, the RT-LAMP method was superior to the other POC testing methods. It was found to be promising, quick, easy to use, and highly efficient. RT-LAMP may alter COVID-19 recognition; these particular RT-LAMP assays for SARS-CoV-2 detection are both fast and accurate.

Currently, a chip-based RT-PCR test is used for detecting Beta CoV and RdRp genes in India. However, a positive screening test requires a second Rdrp gene confirmatory test. Another significant milestone is the reporting of asymptomatic or very mild cases of infection, making it difficult to determine the accurate or exact number of infected people in the population. The IgG-IgM combined antibody test has proven to be more effective in detecting antibody prevalence among infected people. This type of test usually takes less than 15 minutes and can tell if the patient has had a recent or previous SARS-CoV-2 infection.

## Acknowledgments

We are thankful to Cansu İlke Kuru from the Department of Biochemistry, Faculty of Science, Ege University and Buca Municipality, Kızılçullu Science and Art Center, Izmir, Turkey and Fulden Ulucan from the Department of Bioengineering, Ege University, Bornova, Izmir, Turkey for their help in retrieved the data.

## Conflict of interest

The authors declare that there is no conflict of interest.
